# *Dermatobia hominis* in a dog imported from Brazil to Romania

**DOI:** 10.1186/s13071-020-04264-2

**Published:** 2020-07-30

**Authors:** Georgiana Deak, Angela Monica Ionică, Giulia Nădășan-Cozma, Andrei Daniel Mihalca

**Affiliations:** 1 Department of Parasitology and Parasitic Diseases, University of Agricultural Sciences and Veterinary Medicine of Cluj-Napoca, Calea Mănăștur 3-5, 400372 Cluj-Napoca, Romania; 2 Molecular Biology and Veterinary Parasitology Unit (CDS 9), “Regele Mihai I al Romaniei” Life Science Institute, University of Agricultural Sciences and Veterinary Medicine of Cluj-Napoca, Calea Mănăștur 3-5, 400372 Cluj-Napoca, Romania; 3 Vetpoint Vest Arad Private Clinic, Calea Radnei 113, 310269 Arad, Romania

**Keywords:** *Dermatobia hominis*, Imported dog, Romania

## Abstract

**Background:**

*Dermatobia hominis* (Diptera: Oestridae: Cuterebrinae) is a parasite with an important zoonotic and economical impact in the cattle industry, distributed in Central and South America, inhabiting wooded areas along rivers and lowlands. It infests mammals including humans. Lately, there has been a growing trend for people to travel on holidays with their pet dog and also international trade of dogs has increased significantly in the last two decades. Hence, the risk of importation of exotic parasites, including agents of myiasis has increased. *Dermatobia hominis* has been commonly reported as an imported parasite to various countries, mostly as human cases and currently there are only two published cases of *D. hominis* imported with dogs to Europe. Herein, we report a case of *D. hominis* infestation in Romania in a dog recently imported from Brazil.

**Methods:**

Larvae were manually extracted from nodules of a 4-month old non-neutered male, Fila Brasileiro in Arad, Romania. The larvae were morphologically identified, and one specimen was characterized molecularly by amplification and sequencing of a fragment of the mitochondrial cytochrome *c* oxidase subunit 1 gene (*cox*1).

**Results:**

All larvae were morphologically identified as L3 of *Dermatobia hominis.* The BLAST analysis revealed a 98.81% nucleotide similarity to two *D. hominis* isolates from Brazil. The sequence was deposited in the GenBank database under the accession number MT364820.

**Conclusions:**

The travel history of dogs is an important part of the veterinary anamnesis questions and should be thoroughly conducted in the daily practice. Also, prior to and after the importation of dogs from tropical regions, a thorough check of the body surface to detect the presence of nodules is recommended. 
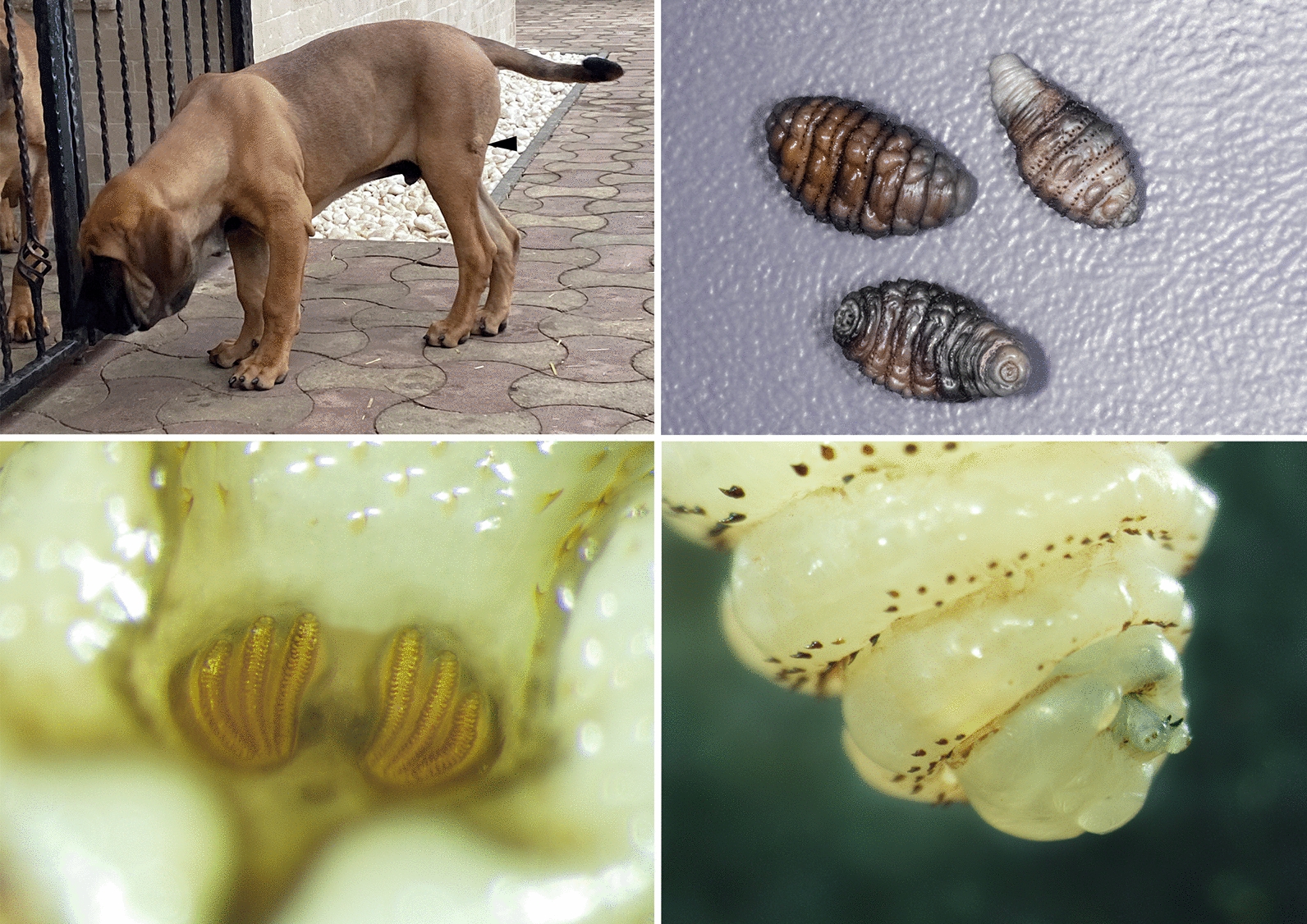

## Background

The genus *Dermatobia* (Oestridae: Cuterebrinae) includes a single species, *Dermatobia hominis* (Linnaeus, 1781) known as “berne”, “torsalo”, “the human botfly”, “ver macaque”, or “tropical warble fly” and affects a wide variety of hosts in the Neotropical region. Its main impact is in the cattle industry and it is also a relevant agent of human myiasis. Other hosts, including dogs are considered accidental. *Dermatobia* larvae feed on tissues or body liquids and are typically responsible for a diverse range of pathology, but mainly causing cutaneous lesions. They are agents of obligatory myiasis and depend on their host to complete the life cycle, producing furuncular lesions [1, 2].

The human botfly is distributed in Central, South and southern North America, inhabiting wooded areas along rivers and lowlands. It typically infests mammals including humans and rarely birds [3–5].

Lately, pet owners travel more and more frequently with their dogs [6, 7]. Moreover, it has been suggested that the international trade with dogs has increased in the last two decades, including dogs from tropical countries [8]. Hence, the risk of importation of exotic parasites, including agent of myiasis is increased. *Dermatobia hominis* has been commonly reported as an imported parasite to various countries, mostly as human cases [9–11]. However, cases of importation with infested dogs are not so commonly reported. Currently, there are only two published cases of *D. hominis* imported with dogs to Europe, one in France [12] and one in the Netherlands [13]. Here, we report a case of *D. hominis* infestation in Romania in a dog recently imported from Brazil.

## Methods

**Fig. 1:**
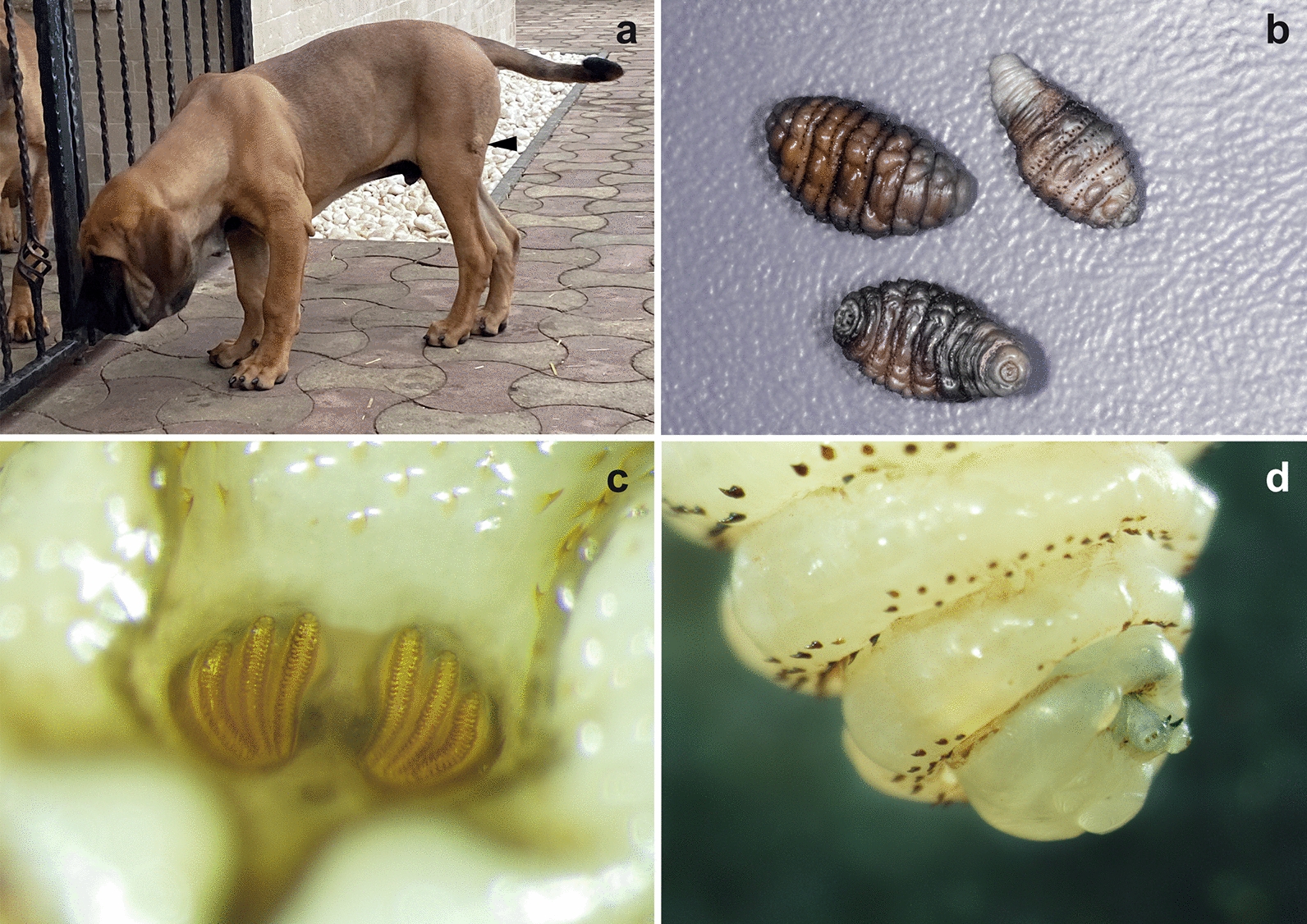
**a** A nodule is visible on the lateral side of the thigh (arrowhead). **b** Larvae (L2 and L3) of *Dermatobia hominis* after extraction from the nodules with the typical narrowed posterior extremity. **c** The typical shape of the spiracles with three curved slits placed in a shallow cavity. **d** Anterior extremity of a L2 larva with the presence of buccal maxillae and the posteriorly oriented spines

A 4-months-old non-neutered male, Fila Brasileiro, was referred to a private clinic in the city of Arad, Romania on 22 February 2020 due to the presence of cutaneous nodules on different parts of the body (Fig. 1a). The dog was imported from Brazil (periurban kennel in Itanhandu, State of Minas Gerais) one month before the clinical evaluation. The owner reported a low body score condition (BSC) when the dog arrived in Romania, but it recovered rapidly and was in a good general health. At the time of examination, its appetite and water consumption were normal. Seven days prior to the visit to the clinic, the owner noticed the presence of four nodules: two on the left lateral abdomen, one on the lateral left thigh and one in the interdigital area of the right posterior limb. At that time, the nodules were just small tumefactions of 5–10 mm in diameter. Initially, the dog was treated at another clinic with amoxicillin and clavulanic acid for several days, without any effect on the nodules. At the time of consultation, the nodules were larger (1.5–2 cm), well defined, ulcerated, and erythematous, but not painful and without purulent exudate. The regional lymph nodes were not enlarged. Since its importation to Romania the dog had not been administered any antiparasitic drugs, but, according to the owner and the clinical records, in Brazil the dog was treated 5 times with Vetmax^®^ Plus (fenbendazole, pyrantel pamoate and praziquantel) every 21 days, and with a single application of Frontline^®^ (fipronil) before leaving the country. In the same household as the imported dog, there were three other Fila Brasileiro dogs also imported from Brazil (several years before), one Hovawart dog imported from Germany and several European shorthair cats. None of the other animals had any cutaneous nodules.

As there were no signs of pain, the larvae were manually extracted without sedation. Only local treatment with chlorhexidine 4% (Vetexpert) was applied after extraction. The larvae were stored in absolute ethanol and sent to the Department of Parasitology and Parasitic Diseases (Faculty of Veterinary Medicine Cluj-Napoca) for morphological and molecular identification.

The larvae were identified using the morphological descriptions given by Mathison & Pritt [14].

One specimen was subjected to genomic DNA isolation, using a commercially available kit (Isolate II Genomic DNA Kit, Bioline, London, UK) and molecularly characterized by amplification and sequencing of a fragment of the mitochondrial cytochrome *c* oxidase subunit 1 gene (*cox*1), using the universal invertebrate primer pair LCO/HCO, as previously described [15]. The sequence was compared to those available in GenBank^®^ by Basic Local Alignment Search Tool (BLAST) analysis.

## Results

All larvae were morphologically identified as L3 of *D. hominis*. The larvae were 15–18 mm long, with an oval/fusiform shape, but narrower at the posterior extremity (Fig. 1b). Their body presented flower-like anterior spiracles with backward orientated spines that encircled the thorax. The posterior spiracles had three curved slits which were placed in a shallow cavity (Fig. 1c) and the cuticular spines were absent from the last abdominal segments. The anterior extremity had two prominent mouth hooks or buccal maxillae which serve for fixing and feeding on the host (Fig. 1d).

The BLAST analysis revealed a 98.81% nucleotide similarity to two *D. hominis* isolates from Brazil (GenBank: JQ246701 and AY463155). The sequence was deposited in the GenBank database under the accession number MT364820. The nodules were completely gone after 1 week when the dog was re-checked after the extraction of the larvae.

## Discussion

*Dermatobia hominis* is a common agent of myiasis of humans and animals in Latin America, but in Europe the cases are sporadic and all of them involve travel history [12, 13, 16, 17]. Most of the reports from European countries concern humans who traveled to Central or South America [18, 19], and only two cases of *D. hominis* myiasis cases have previously been reported in dogs from Europe [12, 13]. In Romania, *D. hominis* was reported only once, in 1994, in a 54-year-old female human patient who returned from California [16]. However, as *D. hominis* is not known from California, this report might either refer to another myiasis (most probably *Cuterebra*) or the possible site of infection was wrongly identified.

*Dermatobia hominis* has an interesting life-cycle, as the eggs can be phoretically transported to the body surface of the host by various hematophagous insects (most commonly mosquitoes) where the L1 hatch and actively penetrate the skin. It takes 5–10 weeks until larvae develop to L3, emerge from skin, and drop to the ground to pupate, and afterwards emerge as adults [20].

In humans, the most effective treatment is the surgical removal of larvae from nodules, or their suffocation using occlusive substances to the opening in the nodules through which the larva breathes [21]. Currently, there is no licensed drug for the treatment or prevention of the infestation with *Dermatobia* in dogs. In humans, oral ivermectin has been successfully used to treat cases where larvae are located in inaccessible regions of the body [22].

## Conclusion

The travel history of dogs is an important part of the veterinary anamnesis questions and should be thoroughly conducted in the daily practice. The travel medicine of pets is an increasingly important field of veterinary medicine. Prior to and after the importation of dogs from tropical regions, to decrease the risk of myiases, we recommend a thorough check of the body surface of dogs to detect the presence of nodules.

## Data Availability

Data sharing is not applicable to this article as no datasets were generated or analyzed during the present study.
